# Reproducibility of small Ge_2_C_6_H_10_O_7_-added MgB_2_ bulks fabricated by ex situ Spark Plasma Sintering used in compound bulk magnets with a trapped magnetic field above 5 T

**DOI:** 10.1038/s41598-020-67463-y

**Published:** 2020-06-29

**Authors:** P. Badica, G. Aldica, M. A. Grigoroscuta, M. Burdusel, I. Pasuk, D. Batalu, K. Berger, A. Koblischka-Veneva, M. R. Koblischka

**Affiliations:** 1 National Institute of Materials Physics, Street Atomistilor 405A, 077125 Magurele, Romania; 2 University Politehnica of Bucharest, Splaiul Independentei 313, 060042 Bucharest, Romania; 3 Fileo Buildup SRL, Street Aleea Politehnicii 4, 060816 Bucharest, Romania; 4 Université de Lorraine, GREEN, 54000 Nancy, France; 5 Experimental Physics, Saarland University, P.O. Box 151150, 66041 Saarbrücken, Germany; 6 Superconducting Materials Laboratory, Department of Materials Science and Engineering, Shibaura Institute of Technology, Tokyo, 135-8548 Japan

**Keywords:** Energy storage, Applied physics, Superconducting properties and materials

## Abstract

Bulk discs (20 mm diameter and 4.3 mm thickness) of MgB_2_ added with Ge_2_C_6_H_10_O_7_ were obtained by Spark Plasma Sintering. Six samples with composition MgB_2_(Ge_2_C_6_H_10_O_7_)_0.0014_ and one undoped sample were fabricated under similar conditions and were magnetically characterized in order to determine the scattering of properties and reproducibility. The main source of the scattering of the properties is the decomposition of the additive due to elimination of the organic part in gas form, which occurs stepwise with intensive vacuum drops at around ~ 560 and ~ 740 °C. A third drop, which is sometimes not well resolved being part of the second peak at 740 °C, occurs at ~ 820 °C. The critical temperature at the midpoint of the transition, *T*_c_, shows only a relatively small variation between 37.4 and 38 K, and the irreversibility field at a low temperature of 5 K takes values between 8 and 10 T. The pinning force and pinning force related parameters do not correlate with the carbon substituting for boron in MgB_2_ and suggest a synergetic influence of the microstructural details and carbon. Overall, despite the superconducting properties scattering, the samples are of high quality. Stacked into a column of six samples, they can trap at the center and on the surface of the column a magnetic field of 6.78 and 5.19 T at 12 K, 5.20 and 3.98 T at 20 K and 2.39, and 1.96 T at 30 K. These promising values, combined with facile fabrication of the samples with relatively high quality and reproducibility, show the feasibility of their use in building complex and large compound arrangements for bulk magnets and other applications.

## Introduction

MgB_2_ is a lightweight practical superconductor (density 2.63 g/cm^3^), hence, it is highly valuable in portable applications. MgB_2_ is a superconductor free of expensive rare earth and precious elements. Considering these aspects, MgB_2_ bulks are of much interest for use in superconducting magnets^1–24^ and related applications. For the development of the applications mentioned, an enhancement of the superconducting functional parameters of MgB_2_ such as the critical current density (*J*_c_), the irreversibility fields (*H*_irr_), and the trapped magnetic field (*B*_tr_) is required. Routes for control and enhancement of the properties in MgB_2_ are related to the use of additions, specifics of the processing technologies roughly classified as in situ and ex situ, and to the quality and features of the raw materials. In the in situ approach, the superconductor is fabricated by reacting Mg and B at temperatures of 650–900 °C, while in the ex situ method the MgB_2_ compound is sintered at higher temperatures up to about 1,200 °C.

In our work we fabricated by ex situ spark plasma sintering (SPS) high density MgB_2_ bulks, pristine and with different additives^25^. Processing conditions were optimized for samples of 2 cm in diameter, i.e. for relatively small ones. Noteworthy is that larger samples (up to 6 cm in diameter in our experiments) exhibit a lower density and less uniformity, hence, this is an undesirable situation since superconducting properties are suppressed.

SPS technique applies a pulsed current on a die system with punches loaded with the MgB_2_ raw powder. The sample is subjected to uniaxial compression. Both, the current and the pressure on the sample facilitate consolidation processes. SPS has a high flexibility allowing high heating and cooling rates and it provides an excellent control of the processing parameters. Despite this, the fabrication of MgB_2_ bulks has some general drawbacks:

(i) Superconducting materials including MgB_2_ are very sensitive to external factors and to achieve a high production reproducibility is not trivial;
(ii) Bulks with complex shapes and large sizes are difficult to fabricate and the scaling process is challenging.

For the MgB_2_ superconductor, the particular reasons are related to the highly volatile nature of Mg, the refractory character of B, the temperature behavior of the additives, the brittle and hard identity of MgB_2_, and the complexity of the processes taking place during sintering, depending on the specifics of the technology. Search for solutions by proper understanding of the processes and their problems in certain conditions is essential for the assessment of the potential for industrial implementation of a given technology and material. An important question is the need of fabricating large-sized samples, e.g., for bulk magnet applications. Assembly of small samples into a desired complex arrangement or geometry instead of using a single large piece can be a simple and convenient solution, if the performance of the superconducting device composed of many elements is high and the fabrication costs are acceptable. The use of small pieces can also overcome the problem of building not only large, but also complex shapes that are otherwise difficult or impossible to fabricate. The addressed aspects are among the key priorities of the research in the applied superconductivity community and through the study proposed in this work, we provide several arguments in favor of small bulks and for their use in compound magnets and other applications.

Many additives introduced to MgB_2_ that is fabricated by ex situ SPS improve the superconducting functional parameters^25^. Among them, the organometallic addition of Repagermanium (Ge_2_C_6_H_10_O_7_) greatly enhances the pinning force, leading to higher *J*_c_ and *H*_irr_. However, its use is expected to pose extra difficulties associated with its decomposition and reaction with MgB_2_ in the SPS processing. The thermal behavior of Ge_2_C_6_H_10_O_7_ and of MgB_2_ added with Ge_2_C_6_H_10_O_7_ was investigated by DSC/DTA/TG/mass spectroscopy measurements and the results were previously reported in Refs.^26,27^. In this work, we explore the reproducibility details from the structural, magnetic and pinning force viewpoints, when samples with the starting composition MgB_2_(Ge_2_C_6_H_10_O_7_)_0.0014_ are processed under fixed, optimized SPS conditions and the initial sample weight is kept constant. As-fabricated samples are disc-shaped with a diameter of 2 cm and a thickness of ~ 4.3 mm. They are defined as ‘small’ bulks. Six samples with additive and one pristine sample are compared with each other. The scattering of the superconducting properties is revealed and discussed in detail. Overall, the sample quality is high and this promotes their use for applications. Considering that a higher volume of the superconductor should generate larger magnetic fields^6,16,20^, we designed and performed an experiment to investigate the trapped field of all six added MgB_2_ discs stacked together in a column of about 27 mm height. The measured values of the trapped magnetic field on the surface of the column are larger than 5 T at 12 K, and 4 T at 20 K. This compound device shows good thermal stability when the decrease rate of the DC applied magnetic field for excitation in the + 5 to − 5 T range is 0.01 T/min. Results infer that the small MgB_2_ bulks added with Ge_2_C_6_H_10_O_7_, as individual pieces or combined into complex arrangements have potential for magnet and related applications: the fabrication of large MgB_2_ samples is not mandatory and small, high quality bulk samples can be employed.

## Methods

The raw materials (Alfa Aesar) were MgB_2_ powder (99.5% metal basis), and Ge_2_C_6_H_10_O_7_ (Repagermanium, Ge-132) (99.7% purity). Powder mixtures of 3.564 g (3.5275 g of MgB_2_ and 0.0365 g of Ge-132) were loaded into graphite dies. Dies were introduced in a SPS furnace produced by FCT Systeme GmbH, Germany (model HPD-5). Six samples with additions and one pristine sample were processed by SPS at 1,150 °C for 3 min and under a uniaxial maximum pressure of 95 MPa^28^. The initial vacuum pressure in the SPS furnace was about 40 Pa. The heating rate was 110 °C/min. The details of the sintered discs are presented in Table 1.

**Table 1:** Samples, lattice parameters *a* and *c*, amount *y* of carbon substituting boron in MgB_2_ (Mg(B_1−*y*_C_*y*_)_2_), phase content, crystallite size, residual strain, apparent density, and midpoint critical temperature *T*_c_^midpoint^.

Sample	Lattice parameters of MgB_2_		*y*	Phase content (wt%)/crystallite size (nm)				Strain in MgB_2_ (%)	Apparent density *ρa*^*SPS*^ (g/cm^3^)	*T*_c_^midpoint^ (K)
	a (Å)	c (Å)		MgB_2_	MgB_4_	MgO	Mg_2_Ge			
I (pristine)	3.0836 ± 0.0003	3.5276 ± 0.0008	0.007 ± 0.001	78.6 ± 0.5/113 ± 3	13.3 ± 0.3/99 ± 3	8.1 ± 0.2/31 ± 1	–	0.14	2.51 ± 0.02	38.2
II	3.0812 ± 0.0007	3.5321 ± 0.0003	0.012 ± 0.002	73.2 ± 0.5/142 ± 2	16.1 ± 0.3/71 ± 3	10.0 ± 0.1/65 ± 1	0.70 ± 0.03/56 ± 5	0.15	2.57 ± 0.02	37.4
III	3.0824 ± 0.0008	3.5254 ± 0.0005	0.010 ± 0.002	73.5 ± 0.5/158 ± 4	16.1 ± 0.3/73 ± 1	9.7 ± 0.1/76 ± 2	0.70 ± 0.03/56 ± 5	0.14	2.55 ± 0.02	37.4
IV	3.0811 ± 0.0005	3.5260 ± 0.0004	0.012 ± 0.001	74.5 ± 0.5/154 ± 4	15.4 ± 0.3/71 ± 3	9.4 ± 0.1/74 ± 2	0.75 ± 0.03/60 ± 5	0.14	2.58 ± 0.02	37.4
V	3.0806 ± 0.0002	3.5248 ± 0.0009	0.013 ± 0.001	73.0 ± 0.5/158 ± 3	16.4 ± 0.3/72 ± 1	10.0 ± 0.1/72 ± 2	0.70 ± 0.03/61 ± 6	0.15	2.58 ± 0.01	37.5
VI	3.0801 ± 0.0006	3.5249 ± 0.0008	0.015 ± 0.001	72.6 ± 0.5/154 ± 3	16.6 ± 0.3/70 ± 2	10.0 ± 0.1/73 ± 2	0.75 ± 0.03/59 ± 3	0.14	2.57 ± 0.01	37.6
VII	3.0813 ± 0.0005	3.5254 ± 0.0008	0.012 ± 0.001	74.4 ± 0.5/149 ± 3	15.3 ± 0.3/73 ± 5	9.6 ± 0.2/75 ± 2	0.70 ± 0.10/57 ± 4	0.14	2.60 ± 0.01	37.4

The apparent densities *ρa*^*SPS*^ (Table 1) of the SPSed discs were measured by the Archimedes method in toluene. The theoretical densities *ρt*^*SPS*^ of the composites were determined according to Ref.^29^ considering the phase assembly. The samples are composed of MgB_2_ (2.63 g/cm^2^), MgO (3.58 g/cm^2^), MgB_4_ (2.49 g/cm^3^), and Mg_2_Ge (2.61 g/cm^2^). The relative density is *R*^*SPS*^ = *ρa*^*SPS*^/*ρt*^*SPS*^ × 100%.

X-ray diffraction (XRD) patterns were measured employing a Bruker AXS D8 Advance diffractometer (Cu_Kα_ radiation). The weight fraction of the phases (Table 1) was extracted by Rietveld analysis (MAUD 2.31^30^). The lattice parameters, *a* and *c* of MgB_2_, the crystallite size, and the residual strain for different phases were estimated (Table 1). The amount of carbon, *y*, substituting boron in the crystal lattice of MgB_2_ (Mg(B_1−y_C_y_)_2_) was calculated as a function of *a* (nm), *y* = − 21.9·*a* + 6.76^31^, using data from Refs.^32–34^.

The displacement curves of the punches and the pressure variation in the furnace were registered by the SPS equipment during processing.

Samples were cut from the MgB_2_ disks in the form of parallelepipeds (~ *L* × *l* × *g* = 1.5 × 1.5 × 0.5 mm^3^).

Magnetic hysteresis loops, *m*(*B*), at different temperatures were measured employing a Vibrating Sample Magnetometer (VSM-9T, Cryogenic). The critical current density, *J*_c_, was determined with the Bean formula for a plate-like geometry^35^:

1 $$J_{{\text{c}}} = { 2}0 \cdot \left| {m \uparrow - m \downarrow } \right|/\{ V \cdot l \cdot [{1} - l/({3} \cdot L)]\} ,$$

where *m* is the magnetic moment in emu for ascending and descending magnetic field, *V* is the sample volume in cm^3^, and *L*, *l* are in cm.

The self-field *J*_c0_ (in A/cm^2^) was estimated from the modified Bean relation, considering the descending branch of the hysteresis loop^36^:

2 $$J_{{\text{c}}} = { 6}0 \cdot \left| {m \downarrow } \right|/(V \cdot l).$$

This approach is useful to avoid the complications with macro flux jumps that occur at low temperatures and with the estimation of |*m*↑ − *m*↓| in the classic Bean model. The irreversibility field μ_0_*H*_irr_ was established for a criterion of 100 A/cm^2^.

The pinning force (*F*_p_ = *J*_c_ × µ_0_⋅*H*_appl_) was calculated. Fitting of the experimental data with the *universal scaling law*^37,38^

3 $$f_{{\text{p}}} = {\text{ A}} \cdot h^{p} \cdot \left( {{1} - h} \right)^{q} ,$$

where *f*_p_ = *F*_p_/*F*_p,max_ with *F*_p,max_ being the maximum pinning force, *h* = *H*_appl_/*H*_irr_ , and *A*, *p* and *q* are fitting parameters, provides in the case of MgB_2_^25^ *p* and *q* values far away from the theoretical ones. From this reason we shall not present them here. Considering percolation aspects, Eisterer^39^ defined the parameter *k*_n_ = *h*_0_/*h*(*f*_p_/2). The values of *h*_0_ = *h*(*f*_p_ = 1) and *k*_n_ provide information on the vortex pinning mechanisms^38^. Theoretical values for Grain-Boundary Pinning (GBP) and Point Pinning (PP) are *h*_0_ = 0.2, *k*_n_ = 0.34 and *h*_0_ = 0.33, *k*_n_ = 0.47, respectively^38,39^.

**Figure 1:**
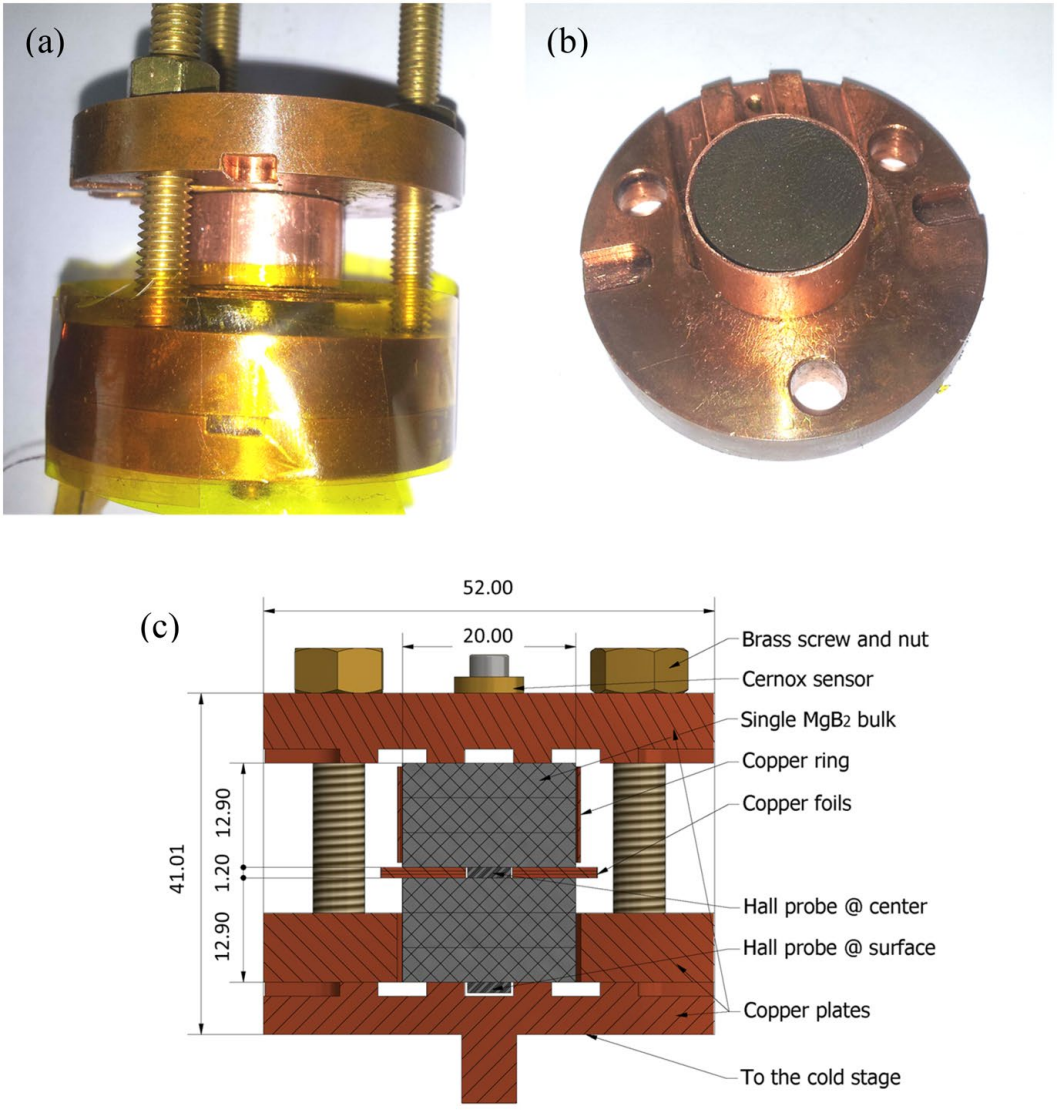
(**a**) Sample stage for trapped magnetic field measurement; (**b**) Copper ring with a diameter of 20 mm in which are introduced the MgB_2_ superconducting discs for a rigid assembly into the compound column-magnet stack; (**c**) Schematic drawing (cross-section) of the six discs mounted in the sample holder. All dimensions are in mm.

Trapped magnetic field experiments were performed by using a 5 T superconducting coil (Oxford Instruments) built in a low-loss cryostat. The room-temperature bore of the coil has a diameter of 75 mm and a cryocooler ARS-4K system is fitted inside it. The lowest temperature that can be used for the measurements is about 9 K. A bi-directional power supply for the superconducting coil (Oxford Instruments) enables a continuous sweep of the magnetic field with rates up to 2.4 T/min (40 mT/s). Hall probes with a linear signal and with a sensibility higher than 70 mV/T are of HHP-NP type from Arepoc s.r.o. (Slovakia). The size of the Hall sensor is 7 × 5 × 1 mm^3^ and the size of the active area is 0.35 mm^2^. In our experiments we use simultaneously two sensors: one is placed inside the column made of 6 MgB_2_ added discs, and the other one is located on the surface of the stacked column. The sample stage with the mounted samples is presented in Fig. 1a, c. The Hall probe ‘at the center’ corresponds to the situation of a pair-type magnet where each magnet is formed by three stacked discs of MgB_2_. A copper ring (Fig. 1b) is fastened around the upper 3-samples stack to provide additional rigidity and to improve the thermal transfer during the cooling of the samples. For thermal purpose, copper foils are placed in between the ‘pairs’. They also play the role of spacers for the introduction of the Hall probe. Two Cernox sensors, one on the cryocooler cold head and the second one on the opposite side of the sample holder measure the minimal *T*_min_ and maximal *T*_max_ temperatures. The temperature of the MgB_2_ stacked column is taken as the average of the two temperatures. The difference between the two temperatures Δ*T* = *T*_max_ − *T*_min_ is 10, 6 and 4 K for a mean temperature of 12, 20 and 30 K, respectively. Samples are energized in the *field-cooling* process: in the normal state, e.g. at 40 K, a magnetic field of 5 T is applied, and the sample is cooled down to the desired temperature. Then, the applied magnetic field, *B*_app_, is decreased with a fixed sweep rate towards − 5 T. The sweep rate is selected as high as possible, but so that the thermomagnetic instabilities (macro flux jumps) are avoided. The difference between the measured field, *B*_meas_, and the applied field, *B*_app_, defines the trapped magnetic field *B*_tr_. In the literature^35^, often the trapped magnetic field is taken for *B*_app_ = 0 T. Actually, this is the remnant trapped magnetic field *B*_rem_ = *B*_tr_ (0 T) and considering the magnetic polarization *µ*_0_*M*, the maximum trapped value is higher and it is obtained at negative values of the sweeping applied magnetic field *B*_app_. We shall consider and use the notation, *B*_tr.max_, for the maximum value of the trapped magnetic field. The local density of magnetic energy *W* is calculated as:

4 $$W = B_{{{\text{meas}}}} \cdot H_{{{\text{app}}}} .$$

## Results

### Processing and evolution of structural details during SPS

**Figure 2:**
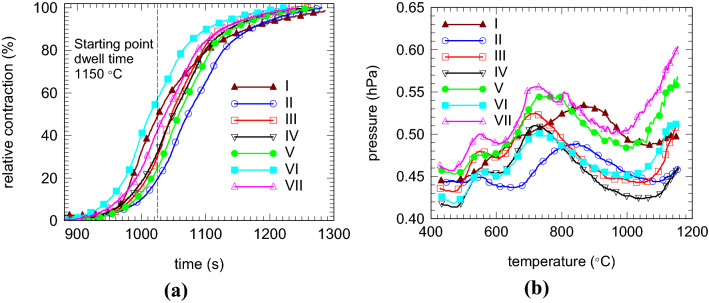
The evolution of the relative contraction with time (**a**), and the pressure in the SPS furnace as a function of temperature (**b**) for samples I–VII during the SPS processing.

The curves showing the relative contraction of the samples during SPS determined from the vertical displacement of the die punches as a function of processing time are presented in Fig. 2a. The curves for the samples II–VII with addition present some scattering, but the shape of the curves is found to be roughly similar. The shape of the curve of the pristine sample I is clearly different, showing a slower saturation on advanced stages of the SPS processing. The apparent density of the pristine sample I (2.51 g/cm^3^, Table 1) is slightly lower than for the samples with additions II–VII (2.55–2.60 g/cm^3^, Table 1). Results suggest that the additive has a strong influence on the densification processes during SPS, promoting a higher apparent density of the sintered bulk. The scattering of the apparent density versus theoretical density of added MgB_2_ samples is (2.60–2.55)/2.63 × 100 = 1.9%. This value is appreciated as acceptably low considering that a relative density above 90% as for our samples from this work has a very low influence on the critical current density^28,40^.

Scattering of the density and of the superconducting properties addressed in “Methods” are linked with reproducibility of the SPS processes, critical being those in which gas phases participate. Contribution is from Mg in the gas state and from the Ge-132 additive thermally decomposing with elimination of the organic component in the gas form. According to Mg-B phase diagrams from Ref.^41^, under our SPS processing conditions, the presence of Mg in the gas state is expected. Mg occurs as the result of the decomposition reactions:

5 $${\text{MgB}}_{{2}} \to {\text{Mg }} + {\text{ 2B,}}$$

6 $${\text{2MgB}}_{{2}} \to {\text{Mg }} + {\text{ MgB}}_{{4}} .$$

**Figure 3:**
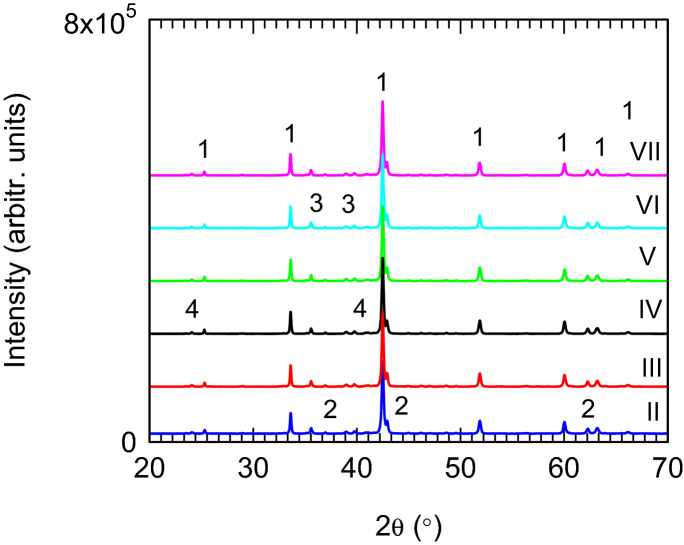
XRD patterns of samples “II–VII” (Table 1). Phases are 1-MgB_2_ (ICDD 72-7019), 2-MgO (ICDD 35-0821), 3-MgB_4_ (ICDD 73-1014), and 4-Mg_2_Ge (ICDD 02-1135).

Free boron from (5) was not present in our XRD patterns. B-based phases in the Mg-B-O system from MgB_2_ samples are observed by electron microscopy and due to their nanometer size, these are not detected by XRD. Reaction (6) is justified by the presence of MgB_4_ phase in our XRD patterns (Fig. 3, Table 1).

The gas pressure in the SPS furnace during SPS processing has a complex behavior. For the samples with additions II–VII, intensive vacuum drops (or peaks of pressure-increase in the furnace in Fig. 2b) are observed at around ~ 560 and ~ 740 °C. A third peak that sometimes is not well resolved (being part of the second peak at 740 °C), occurs at ~ 820 °C. Its intensity is lower than for the peak at ~ 740 °C (an exception is seen for sample II). In the pristine sample, the first peak does not occur, the second one is very broad, and its maximum is found at ~ 840 °C. Therefore, the peaks at ~ 560 and 820 °C are ascribed to processes with gas formation. The first peak is generated mainly by decomposition of Ge-132 in the added samples, and the second is related with MgB_2_ behavior since it occurs in the pristine sample. In addition, we note that in Ar atmosphere and for a heating rate of 10 °C/min, Ge-132, pristine or in a powder mixture with composition (MgB_2_)_0.97_(Ge_2_C_6_H_10_O_7_)_0.03_, starts decomposing at low temperatures ~ 215 °C^26,27^. In experiments from Ref.^27^, the crystalline Ge and MgO were detected before formation of Mg_2_Ge and MgB_4_, when the temperature approaches the melting point of Ge (938 °C). This implies that the reaction (6) occurs at higher temperatures than reaction (5) and Mg as a product of (6) easily reacts with residual oxygen and forms MgO:

7 $${\text{Mg }} + { 1}/{\text{2O}}_{{2}} \to {\text{MgO}}{.}$$

The Ge-metal from the Ge-132 decomposition reacts with Mg from (5) and especially from (6) by the following reaction:

8 $${\text{2Mg }} + {\text{ Ge}} \to {\text{Mg}}_{{2}} {\text{Ge}}{.}$$

Carbon substitution for boron in the crystal lattice of MgB_2_ was observed above 590 °C.

The results presented here cannot be directly applied to processes during SPS since in this case the atmosphere is vacuum, the heating rate is much higher (110 °C/min) and a uniaxial pressure is applied to the sample during SPS, but they are helpful to understand the reaction mechanisms.

For SPS conditions, the use of Ge-132 additive influences the reactions (5)–(7): the amount of MgB_2_ in the pristine sample (78.6 wt%) is higher than in the samples with addition (72.6–74.5 wt%) and the difference (4.1–6 wt%) is larger than the amount of Mg_2_Ge (0.7–0.75 wt%). Thus, the additive promotes the decomposition of MgB_2_ and the reactions (5 + 7) and (6), but the features of these reactions comparative to pristine sample are modified. In support of this idea we also note that in the added samples II–VII the crystallite size of MgO (65–76 nm) is larger and the size of the MgB_4_ grains is smaller (70–73 nm) than in the pristine sample (31 and 99 nm, Table 1). Moreover, the average crystallite size of MgB_2_ in the added samples (146–158 nm) is larger than for the pristine sample (113 nm).

Another observation of interest is that in the samples with addition, above 1,100 °C, the vacuum in the SPS furnace shows the tendency for worsening (Fig. 2b). A possible decomposition reaction at high temperatures during SPS is:

9 $${\text{MgB}}_{{4}} \to {\text{Mg }} + {\text{ 4B}}{.}$$

The additive is acting also as a source of carbon substituting boron in MgB_2_. Possible mechanisms involved in this process might be similar to those for other organic type compounds^42^ added to MgB_2_, but more research is needed. In added samples the amount of C (0.010–0.015) is higher than in the pristine sample (0.007). The *y*_Carbon_ in the raw powder was 0.002. This value is lower than for the pristine sample I (*y* = 0.007). Data suggest that there is an intake of carbon from the graphite die used in the SPS processing. The scattering of *y*_Carbon_-values in added samples is apparently high. Despite of this, the microstrain is not sensitive to the *y*-variation as one would expect, and it takes an approximately constant value of 0.14–0.15%. For samples with addition, no direct correlation was observed between the vacuum pressure behavior during SPS and the structural parameters scattering.

### Critical current density, irreversibility field, and pinning force

**Figure 4:**
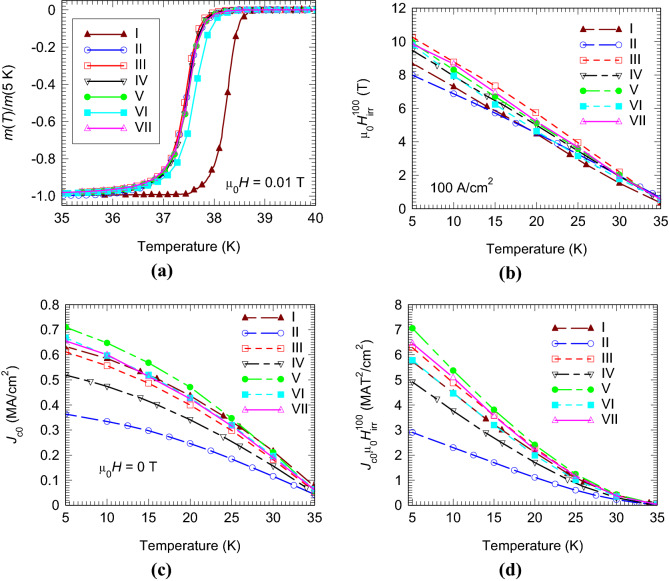
Temperature dependence of the reduced magnetization versus temperature measured in zero-field-cooling arrangement at 0.01 T (**a**), irreversibility field µ_0_*H*_irr_ (**b**), zero-field critical current density *J*_c0_ (**c**), and the product (*J*_c0_ × μ_0_*H*_irr_) for pristine and added samples II–VII (**d**).

A higher amount *y* of carbon in the samples with addition (Table 1), pushes the midpoint critical temperature *T*_c_^midpoint^ (37.4–37.6 K) to lower values than in the pristine sample (38.2 K) (Fig. 4a).

**Figure 5:**
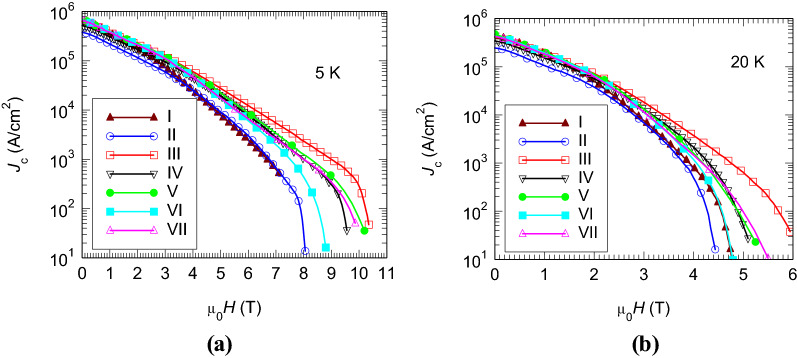
Critical current density versus applied magnetic field for samples I–VII at: (**a**) 5 K and (**b**) 20 K.

Although the decrease is not significant, only about 0.6–0.8 K, the carbon substitution for boron in MgB_2_ and the changes in the composite microstructure of the added samples promote an increase of the critical current density *J*_c_ at high magnetic fields (Fig. 5). The microstructure was reported in Ref.^43^. Filamentary 1D nano grains with Ge and Mg ascribed to Mg_2_Ge of 10–25 nm in length and 2–3 nm thickness were observed. Their size is comparable with coherence length of MgB_2_, and, hence, they can play the role of efficient pinning centers. Also, the other secondary phases, MgO and MgB_4_, form nano precipitates and can contribute pinning and *J*_c_ at high magnetic fields.

The enhancement of *J*_c_ at high magnetic fields is reflected by an increase of µ_0_*H*_irr_ at different temperatures (Fig. 4b) in the added samples (the exception from this behavior is sample II). At 5 K, the enhancement of µ_0_*H*_irr_ is up to 2 T when compared with the value for pristine sample I. Hence, the maximum enhancement, or the scattering of the irreversibility field in added samples represents about 25% of the µ_0_*H*_irr_ for the pristine sample.

A decrease of *J*_c0_ is observed for some of the added samples (e.g. for samples II and IV, Fig. 4c) and it does not correlate with the amount of carbon, *y*: usually, a higher *y* decreases *J*_c0_, but this is not the case here. The samples V and VI with the highest values of *y* (Table 1) show larger and almost similar *J*_c0_ as compared to those for pristine sample I, respectively, in the entire studied temperature range (5–35 K). According to Birajdar and Eibl^44^, ‘colonies’ of MgB_2_ influence *J*_c0_. Larger colonies promote a higher *J*_c0_. The Ge-132 added samples^43^ are composed of Ge poor and rich regions. The Ge-poor regions (5–100 µm) are compact and can be associated with ‘colonies’ embedded in a Ge-rich ‘matrix’. In close vicinity of Ge, frequently oxygen was detected. Colonies can be considered relatively clean regions, while the matrix is relatively dirty. The Ge, O, and C distributions and phase assembly contribute to colonies features and *J*_c0_ scattering. Carbon in MgB_2_ is present in the crystal lattice of MgB_2_, as quantified by *y*_Carbon_, and it can be also at grain boundaries. In the second case it is not detected by XRD. The *J*_c0_ variation is relatively low and it takes values at 5 K between 0.36 and 0.71 MA/cm^2^.

The variation of *J*_c0_ among the samples influences the behavior of the quality product (*J*_c0_⋅µ_0_*H*_irr_) (Fig. 4d). The product for samples III, V and VII is higher, for sample VI is similar, and for samples II and IV is lower than the values for pristine sample I. The product is used in our comparative analysis to provide a quantitative measure of quality with information about the ‘balance’ between the superconducting parameters at low and high magnetic fields. The product has no practical or theoretical meaning.

Results from the previous paragraphs suggest that scattering of *J*_c_ and *H*_irr_ in the added samples are induced not only by variation in the carbon amount *y*, but also by microstructural aspects of the composite samples. Both pinning and grain connectivity are influenced. For effective pinning one has to consider the nano precipitates of secondary phases, the density and type of interfaces and grain boundaries, the defects, and the micro strain. For a deeper understanding we shall look on the pinning force features.

**Figure 6:**
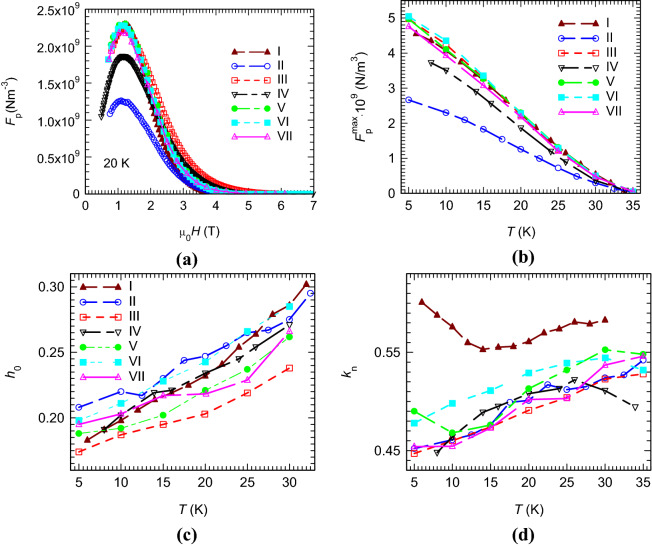
(**a**) The pinning force versus applied magnetic field at 20 K; (**b**) the maximum pinning force vs. temperature; (**c**) the reduced magnetic field for maximum pinning force as function of temperature; (**d**) pinning-force-related parameter *k*_n_ as a function of temperature. The samples notation is as given in Table 1 (I is the pristine sample and II–VII the added samples).

The added samples II–VII show a variation of the maximum pinning force at 20 K from 1.3 × 10^9^ to 2.3 × 10^9^ Nm^-3^ (Fig. 6a). The maximum pinning force for the pristine sample is, within the error range, similar to the values for the added samples III, V–VII, and it is higher than for samples II and IV at temperatures below 20 K (Fig. 6b). The pinning force parameters, *h*_0_ and *k*_n_ as a function of temperature are presented in Figs. 6c, d.

At low temperatures, all samples show *h*_0_ values close to 0.2, which is apparently suggesting a major grain boundary pinning (GBP) mechanism. At higher temperatures, *h*_0_ increases to values close to 0.3 and indicates a stronger pinning mechanism on point defects (PP). The tendency of *k*_n_ to increase with temperature supports the results of *h*_0_. Nevertheless, the values of *k*_n_ in the entire studied temperature range (5–35 K) are higher than 0.44, being above the theoretical values 0.34 for GPB and close or higher than 0.47 for PP. The contribution of GBP, that is more important at low temperatures, has a limited effect when compared to the predominant PP mechanism. We shall also observe that, as already reported in Ref.^25^, a higher carbon amount *y* has a strong influence and pushes the curves of *h*_0_(*T*) and *k*_n_(*T*) to lower values, regardless of temperature (compare results for pristine sample I and added samples II–VII in Table 1 and in Figs. 6c, d), i.e., the GBP becomes stronger. On the other hand, the influence of nano precipitates and of other microstructural details (grain boundaries, defects, micro strain, others) on pinning force parameters cannot be neglected.

The scattering of *h*_0_ for the samples with addition at different temperatures is between 0.027 and 0.047, while for *k*_n_ the range is between 0.03 and 0.052.

### Trapped magnetic field

**Figure 7:**
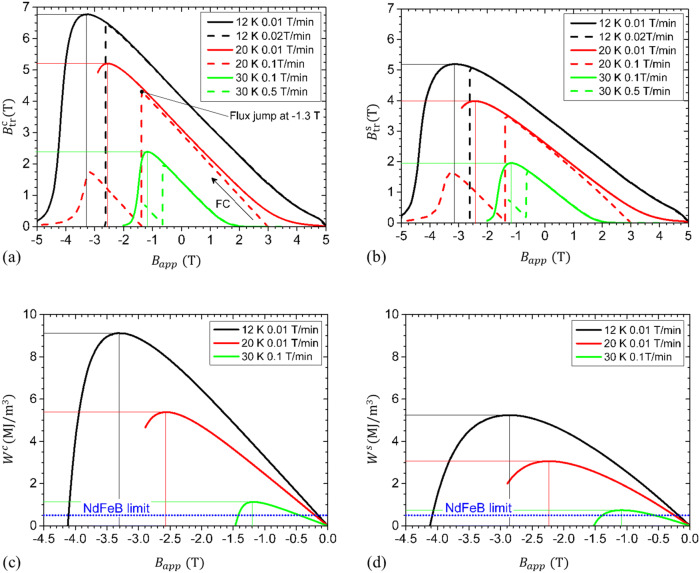
Trapped magnetic field at the center (**a**) and on the surface (**b**) of the magnet composed of 6 MgB_2_ superconducting discs (samples II–VII, Table 1). The local density of magnetic energy at the center (**c**) and on the surface (**d**) is compared with the magnetic energy of a NdFeB conventional magnet.

Trapped magnetic field *B*_tr_ at the center (*B*^c^_tr_) and on the surface (*B*^s^_tr_) of the compound magnet made of 6 discs is presented in Fig. 7. The trapped magnetic field curves without flux jumps were measured at 12, 20 and 30 K for the decrease rate of the DC applied magnetic field for excitation in the + 5 to − 5 T range of 0.01, 0.02, 0.1 and 0.5 T/min, respectively. The lower the working temperature of the compound magnet is, the lower the rate of applied magnetic field sweep without flux jumps should be.

In our work^10^ we measured the MgB_2_-NdFeB pair magnet (at the center) composed of a permanent magnet (thickness 8 mm, diameter 20 mm) placed below of a MgB_2_ superconducting disc (thickness 3.5 mm, diameter 20 mm) fabricated by SPS. The trapped magnetic field attained 3.2 T at 12 K for a sweep rate of the DC applied magnetic field of 0.03 T/min. The NdFeB permanent magnet generates a field of about 0.5 T, while the remnant trapped field *B*_rem_ = *B*_tr_(0 T) of a MgB_2_ disc is about 2.8 T. One observes that the remnant trapped field at the center and at the surface of the column is *B*^c^_rem_ (12 K) = 4.1 T, *B*^s^_rem_ (12 K) = 3.5 T (Fig. 7a, b), i.e., it is higher than for one disc (2.8 T), but it is about 4.1 and 4.8 times lower, respectively, than the multiple of six times (i.e. 16.8 T) of the remnant trapped field for one disc.

The maximum trapped magnetic field of the column at the center and on the surface show high values of 6.78 and 5.19 T at 12 K, 5.20 and 3.98 T at 20 K, and 2.39 and 1.96 T at 30 K. It is remarkable that at 20 K or at lower temperatures the obtained values exceed the application threshold value of 3 T^4^. The maximum trapped field on the surface of our compound magnet (5.19 T) is comparable with record high 5.4 T measured at 12 K in Ref.^6^ on the surface of a single bulk sample (20 mm diameter and 8 mm height) and it is higher than 2.2 T measured at 7.5 K in Ref.^22^ on the surface of a stack of five discs (32 mm diameter, 6 mm thickness).

The values of the maximum local magnetic energy measured at the center *W*^c^ and at the surface *W*^s^ of the compound magnet (Fig. 7c, d) are 2.23 and 1.5 at 30 K, 10.8 and 6.1 at 20 K, and 18.3 and 10.5 at 12 K times larger than the magnetic energy of the NdFeB magnet. The energy of the NdFeB magnet is an intrinsic property of the material and it is not influenced by size and shape.

## Conclusion

Small spark plasma sintered discs of MgB_2_ added with Ge_2_C_6_H_10_O_7_ were obtained for fixed SPS processing conditions and raw materials. They show good reproducibility of the density, structural and superconducting properties. A relatively large scattering was encountered for the irreversibility field suggesting that this parameter is among the most sensitive ones. The scattering of the properties is related to complex decomposition processes of MgB_2_ and of the additive. Contribution arises from carbon substitution for boron in the crystal lattice of MgB_2_, but also from microstructural details.

Six discs were stacked together in a column and measured for trapped magnetic field at the center and on the surface. The trapped magnetic fields at the center and on the surface were 6.78 and 5.19 T at 12 K, 5.20 and 3.98 T at 20 K, and 2.39 and 1.96 T at 30 K, when the decrease rate of the DC applied magnetic field for excitation in the + 5 to − 5 T range was 0.01 at 12 K, 0.01 at 20 K, and 0.1 T/min at 30 K. For the indicated rates, the curves of trapped magnetic field with applied magnetic field are free of flux jumps. Relevant for applications are the values of the trapped field measured on the surface of the compound magnet.

The results enable further studies and use of small MgB_2_ superconducting samples in large and complex-shape compound magnets and other applications. Furthermore, it demonstrates the feasibility of compound magnets made of small and reproducible MgB_2_ discs.
